# Poldip2 mediates blood‐brain barrier disruption and cerebral edema by inducing AQP4 polarity loss in mouse bacterial meningitis model

**DOI:** 10.1111/cns.13446

**Published:** 2020-08-12

**Authors:** Meng Gao, Weitian Lu, Yue Shu, Zhengyu Yang, Shanquan Sun, Jin Xu, Shengwei Gan, Shujuan Zhu, Guoping Qiu, Fei Zhuo, Shiye Xu, Yiying Wang, Junhong Chen, Xuan Wu, Juan Huang

**Affiliations:** ^1^ Department of Anatomy Chongqing Medical University Chongqing China; ^2^ Institute of Neuroscience Chongqing Medical University Chongqing China; ^3^ Department of Neurosurgery The Second Affiliated Hospital Chongqing Medical University Chongqing China

**Keywords:** AQP4, bacterial meningitis, blood‐brain barrier, cerebral edema, MMPs, Poldip2

## Abstract

**Background:**

Specific highly polarized aquaporin‐4 (AQP4) expression is reported to play a crucial role in blood‐brain barrier (BBB) integrity and brain water transport balance. The upregulation of polymerase δ‐interacting protein 2 (Poldip2) was involved in aggravating BBB disruption following ischemic stroke. This study aimed to investigate whether Poldip2‐mediated BBB disruption and cerebral edema formation in mouse bacterial meningitis (BM) model occur via induction of AQP4 polarity loss.

**Methods and Results:**

Mouse BM model was induced by injecting mice with group B hemolytic streptococci via posterior cistern. Recombinant human Poldip2 (rh‐Poldip2) was administered intranasally at 1 hour after BM induction. Small interfering ribonucleic acid (siRNA) targeting Poldip2 was administered by intracerebroventricular (i.c.v) injection at 48 hours before BM induction. A specific inhibitor of matrix metalloproteinases (MMPs), UK383367, was administered intravenously at 0.5 hour before BM induction. Western blotting, immunofluorescence staining, quantitative real‐time PCR, neurobehavioral test, brain water content test, Evans blue (EB) permeability assay, transmission electron microscopy (TEM), and gelatin zymography were carried out. The results showed that Poldip2 was upregulated and AQP4 polarity was lost in mouse BM model. Both Poldip2 siRNA and UK383367 improved neurobehavioral outcomes, alleviated brain edema, preserved the integrity of BBB, and relieved the loss of AQP4 polarity in BM model. Rh‐Poldip2 upregulated the expression of MMPs and glial fibrillary acidic protein (GFAP) and downregulated the expression of β‐dystroglycan (β‐DG), zonula occludens‐1 (ZO‐1), occludin, and claudin‐5; whereas Poldip2 siRNA downregulated the expression of MMPs and GFAP, and upregulated β‐DG, ZO‐1, occludin, and claudin‐5. Similarly, UK383367 downregulated the expression of GFAP and upregulated the expression of β‐DG, ZO‐1, occludin, and claudin‐5.

**Conclusion:**

Poldip2 inhibition alleviated brain edema and preserved the integrity of BBB partially by relieving the loss of AQP4 polarity via MMPs/β‐DG pathway.

## Graphical abstract

Schematic illustration of AQP4 polarity loss being induced by Poldip2 in mouse bacterial meningitis model. Poldip2 induced the upregulation and activation of MMPs, which mediates the cleavage of β‐DG. Due to β‐DG being responsible for AQP4 anchoring to astrocytic endfeet, the degradation of β‐DG results in AQP4 polarity loss. In the present mechanism study, Poldip2 siRNA and the UK383367 were used to inhibit Poldip2 expression and MMP activity, respectively.

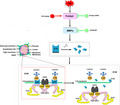

## 1 INTRODUCTION

Bacterial meningitis (BM) is a severe infectious disease with a high morbidity and mortality rate.^1^, ^2^ Despite the advances in antimicrobial therapy for BM, up to 50% of BM survivors develop long‐term neurological sequelae.^3^ One complication of BM is brain edema, which accounts for intracranial hypertension, potentially leading to brain ischemia, herniation, and even death.^4^ Cerebral edema results from fluid accumulation in astrocytes due to energy failure or entry of plasma fluid into the brain extracellular space (ECS) caused by leaky blood‐brain barrier (BBB).^5^, ^6^ The discovery of aquaporins (AQPs), a family of membrane‐bound water channel proteins, contributes to deeper understanding of brain water transport. Aquaporin‐4 (AQP4), the predominant aquaporin in the central nervous system, is expressed widely throughout the brain, particularly at brain‐blood interfaces. AQP4 is abundantly expressed in astrocytic endfeet processes in direct contact with blood vessels.^7^, ^8^, ^9^ The specific highly polarized expression of AQP4 was suggested to play critical roles in maintaining cerebral water balance and BBB integrity.^10^, ^11^, ^12^, ^13^ Previous studies have reported that AQP4 polarity was disturbed in various neurological diseases that cause brain edema and BBB disruption.^14^, ^15^, ^16^, ^17^ However, whether loss of AQP4 polarity mediates BBB disruption and cerebral edema formation in mouse BM model is still unknown.

The polarized distribution of AQP4 relies on dystrophin‐glycoprotein complexes (DGC), a membrane‐spanning group of proteins comprising syntrophin, dystroglycan (DG), utrophin, and dystrobrevins.^18^, ^19^, ^20^ The proteins in DGC connect extracellular matrix (ECM) with cytoskeleton; AQP4 is tethered to syntrophin, a component of DGC; thus, AQP4 is anchored to astrocytic endfeet processes.^18^, ^19^, ^20^ β‐dystroglycan (β‐DG), a membrane‐spanning protein, is the central component of DGC and can be cleaved by matrix metalloproteinases (MMPs).^21^, ^22^ Therefore, we speculate that any stimuli in brain promoting MMPs activity will lead to loss of AQP4 polarity via β‐DG degradation, which further affects the water transport balance in brain and the integrity of BBB.

Polymerase δ‐interacting protein 2 (Poldip2) is a protein discovered originally as a binding partner of the p50 subunit of DNA polymerase δ.^23^ Poldip2 has been shown to be involved in numerous cellular functions such as DNA repair, mitochondrial fusion, cytoskeletal remodeling, and cell proliferation and migration.^23^ A recent study by Hernandes demonstrated that Poldip2 was upregulated following ischemic stroke.^24^ However, changes in Poldip2 expression in BM animal models have not been reported. Furthermore, Hernandes's study reported that Poldip2 mediates the breakdown of BBB by MMPs activation following ischemic stroke,^24^ and another study showed that loss of Poldip2 impaired MMPs activity in a hindlimb ischemic animal model.^25^ Knowing that MMPs cleaved β‐DG and β‐DG is the central protein for AQP4 anchoring to the astrocytic endfeet processes, we induced mouse BM model by injecting mice with group B hemolytic streptococci via the posterior cistern and investigated whether Poldip2 affects AQP4 polarity via the MMPs/β‐DG pathway in this model.

## 2 MATERIALS AND METHODS

### 2.1 Animals

All animal experiments were approved by the Animal Ethics Committee of Chongqing Medical University. A total of 259 male Kunming mice (weighing 20 ± 2 g) were used, of which 25 died during the intracisternal puncture process and were excluded from this study. All mice were housed in an automatically controlled light‐temperature room with free access to food and water.

### 2.2 Bacterial culture

Group B β‐hemolytic streptococci strain was obtained from the microbiology laboratory of Jiulongpo People's Hospital, Chongqing. Bacteria were inoculated on blood agar plates and incubated at 37°C for 16‐24 hours in a 5% CO_2_ incubator. The organisms were isolated at mid‐logarithmic growth phase, washed with saline twice, and diluted to 10^8^ colony‐forming units (CFU)/L.

### 2.3 Mouse bacterial meningitis (BM) model

The BM model was established by bacterial injection as previously described.^26^ Briefly, the mice were anesthetized by intraperitoneal injection of 3.5% chloral hydrate (0.1 mL/10g), and then, 10 μL of cerebrospinal fluid (CSF) was withdrawn from intracisternal puncture followed by intracisternal injection of 10 μL β‐hemolytic streptococcus suspension (10^8^ CFU/L). For the sham animals, instead of bacterial suspension, 10 μL of sterile saline was injected.

### 2.4 Study design

All mice were randomly distributed to the following five separate experiments.

#### 2.4.1 Experiment 1

To determine the time course of endogenous Poldip2 expression after BM induction, mice were randomly divided into seven groups: sham, 3 hours BM, 6 hours BM, 12 hours BM, 24 hours BM, 48 hours BM, and 72 hours BM (n = 6 per group). The brains of the mice were collected for Western blot analysis. In addition, double immunofluorescence staining was performed to test whether Poldip2 is expressed in astrocytes, neurons, microglia, neutrophils, and T cells at 12 hours after BM induction (n = 3 in sham and 12 hours BM group).

#### 2.4.2 Experiment 2

To choose the best Poldip2 siRNA from three candidate sequences (sequences A, B, and C, Figure 2) provided by OriGene (USA), quantitative real‐time PCR and Western blot analysis were performed after BM induction. Mice were randomly divided into four groups: BM + scramble siRNA, BM + sequence A, BM + sequence B, and BM + sequence C (n = 6 per group).

#### 2.4.3 Experiment 3

The effect of Poldip2 siRNA (the best among three candidate sequences for interfering Poldip2 expression) on neurological function, brain water content, BBB permeability, astrocytic swelling, and AQP4 polarized distribution was evaluated by administering Poldip2 siRNA by intracerebroventricular (i.c.v) injection at 48 hours before BM induction. Neurological score assessment, brain water content test, EB permeability assay, transmission electron microscopy (TEM), and immunofluorescence staining experiments were performed after BM induction. Mice were randomly divided into four groups: sham, BM, BM + scramble siRNA, and BM + Poldip2 siRNA (n = 21 per group).

#### 2.4.4 Experiment 4

The downstream proteins of Poldip2 were explored, and the mechanisms underlying Poldip2 effect on BBB integrity were investigated. For this, recombinant human Poldip2 (rh‐Poldip2; Proteintech) and Poldip2 siRNA were used to upregulate and downregulate Poldip2 expression, respectively. The expression levels of MMPs, β‐DG, zonula occludens‐1 (ZO‐1), occludin, claudin‐5, and glial fibrillary acidic protein (GFAP) were determined by Western blotting after BM induction, and the activity of MMPs was evaluated by gelatin zymography. Mice were randomly divided into five groups: sham, BM + vehicle, BM + rh‐Poldip2, BM + Poldip2 siRNA, and BM + scramble siRNA (n = 6 per group).

#### 2.4.5 Experiment 5

To investigate whether MMPs were involved in Poldip2‐mediated BBB disruption and loss of AQP4 polarity in mouse BM model, UK383367 (Selleck), a specific inhibitor of MMPs, was used to inhibit the activity of MMPs. Neurological function, brain water content, BBB permeability, and AQP4 polarity were evaluated by Loeffler's score assessment, brain water content test, EB permeability assay, and immunofluorescence staining experiments, respectively. The expression levels of β‐DG, ZO‐1, occludin, claudin‐5, and GFAP were examined by Western blot. Mice were randomly divided into three groups: sham, BM + vehicle, and BM + UK383367 (n = 24 per group).

### 2.5 In vivo RNAi

Intracerebroventricular (i.c.v) Poldip2 siRNA administration was performed as previously described.^27^ Briefly, mice were anesthetized with chloral hydrate and fixed in a stereotactic frame in prone position. Next, the scalp was sterilized by isopropyl alcohol, and a 10‐μL Hamilton microsyringe was inserted at 1.0 mm anterior and 1.5 mm lateral to the bregma, and 2.5 mm deep from the skull surface into the right lateral ventricle. At 48 hours before BM induction, 2 μL Poldip2 siRNA (100 pmol/μL; OriGene) or scramble siRNA (100 pmol/μL, OriGene) was injected slowly over a period of 5 min. To prevent liquid reflux, the syringe was held in place for 10 minutes and then withdrawn slowly over a period of 5 minutes.

### 2.6 Intranasal administration of rh‐Poldip2

A total volume of 5 μL rh‐Poldip2 (0.3 μg/mice) or vehicle (in sterile saline water) was administered into the bilateral nares. 1.25 μL rh‐Poldip2 or vehicle was administered every 2 minutes into alternating nares.^28^

### 2.7 Intravenous administration of MMPs inhibitor

A total volume of 8 μL UK383367 (4 mg/kg) or vehicle (15% Captisol constituted by PEG 300, Tween‐80, and ddH_2_O) was administered intravenously via the tail vein at 0.5 hour before BM induction.^29^, ^30^

### 2.8 Neurological score assessment

Neurobehavioral functions were assessed by a blinded investigator using Loeffler's scoring method at 12 hours after BM induction, as described previously.^31^ Briefly, the mice were placed on a horizontal floor and the neurobehavioral status of each mouse was recorded. The score criteria of Loeffler's method are as follows: score 1, no movement; score 2, no upright turning when positioned on the back; score 3, upright turning in more than 5 seconds; score 4, minimal ambulatory activity and upright turning in less than 5 seconds; and score 5, normal motor activity and upright turning in less than 5 seconds.

### 2.9 Brain water content

Brain water content was measured at 12 hours after BM induction by dry‐wet weight method as previously described.^15^ Briefly, wet brain was weighed immediately after removal, dried in a vacuum oven at 120°C for 48 hours, and weighed again to determine the dry weight. Brain water content (%) was calculated as [(wet weight − dry weight)/wet weight] × 100%.

### 2.10 Immunofluorescence

Immunofluorescence was performed as previously described.^15^ Briefly, mice were deeply anesthetized and transcardially perfused with 30 mL 0.9% ice‐cold saline followed by 4% paraformaldehyde at 12 hours after BM induction. The brains were removed and fixed in 4% paraformaldehyde overnight at 4°C and then placed in 30% sucrose until they sank. Then, the brains were embedded into optimal cutting temperature (OCT) compound and frozen, and 10‐μm‐thick coronal slices were cut sequentially at − 20°C with a cryostat (CM1860; Leica Microsystems). The prepared slices were permeabilized with 0.3% Triton X‐100 for 30 minutes at 37°C and then blocked with 5% donkey serum for 30 minutes at 37°C. Subsequently, each coronal section was incubated overnight at 4°C with the following primary antibodies: anti‐GFAP (1:200; Cell Signaling Technology, 3670), anti–ionized calcium‐binding adaptor molecule 1 (anti‐Iba‐1, 1:200; Abcam, ab48004), anti–neuronal nuclei (anti‐NeuN, 1:400; Abcam, ab104224), anti‐myeloperoxidase (anti‐MPO, 1:300; Abcam, ab90810), anti‐F4/80 (1:200; Abcam, ab6640), anti‐CD3 (1:200; Abcam, ab33429), anti–early endosome marker (anti‐EEA1, 1:100; Abcam, ab70521), anti‐Poldip2 (1:400; Abcam, ab181841), and anti‐AQP4 (1:400; Abcam, ab46182). The slices were then washed and incubated with the corresponding fluorescence‐conjugated secondary antibodies (1:400, Zsgb BIO) for 1 hour at 37°C. The nucleus was counterstained with 4, 6‐diamidino‐2‐phenylindole (DAPI). The slices were visualized with a fluorescence microscope (IX73, Olympus), and immunoreactivity density was measured using Image‐Pro Plus 6.0 software.

### 2.11 Western blotting

Mice were anesthetized, and the brains were quickly removed and stored in − 80°C freezer. Western blotting was performed as previously described.^32^ Briefly, mice brains were homogenized in radioimmunoprecipitation assay (RIPA) lysis buffer (Beyotime, P0013B) with protease inhibitor cocktail and centrifuged at 14 000 ***g*** for 20 minutes at 4°C. The supernatant was collected, and total protein content was determined using a Bicinchoninic Acid (BCA) Protein Assay Kit (Beyotime, P0010). Protein samples (50 μg proteins/well) were resolved by 10% sodium dodecyl sulfate‐polyacrylamide gel electrophoresis (SDS‐PAGE), and the protein bands were transferred to a polyvinylidene fluoride (PVDF) membrane. The membranes were blocked for 2 hours at 37°C using QuickBlock™ blocking buffer (Proteintech, P0252) and incubated with the following primary antibodies overnight at 4°C: anti‐Poldip2 (1:1000; Abcam, ab181841), anti‐MMP9 (1:1000; Abcam, ab58803), anti‐MMP2 (1:500; Santa Cruz, sc‐53630), anti‐ZO‐1 (1:1000; Affinity, AF5145), anti‐occludin (1:50 000; Abcam, ab167161), anti‐claudin‐5 (1:1000; Abcam, ab15106), anti‐β‐DG (1:500; Santa Cruz, sc‐33702), anti‐GFAP (1:1000; Cell Signaling Technology, 3670), anti–glyceraldehyde 3‐phosphate dehydrogenase (anti‐GAPDH; 1:5000; Proteintech, HRP‐60004), and anti‐β‐actin (1:5000; Proteintech, 20536‐1‐AP). The membranes were then washed and incubated with appropriate secondary antibodies (1:20 000; CST) for 1 h at 37°C. Protein bands were visualized using an enhanced chemiluminescence (ECL) reagent (4A Biotech, 4AW012) and analyzed by Quantity One software (version 4.6.2).

### 2.12 Quantitative real‐time PCR

Total RNA was isolated using the RNAiso Plus reagent (TaKaRa) according to the manufacturer's protocol.^33^ RNA concentration was measured using a NanoDrop 1000 spectrophotometer (Thermo). The total RNA was reverse‐transcribed to cDNA using PrimeScript ^TM^ RT Reagent Kit and gDNA Eraser (TaKaRa). mRNA expression was measured using CFX Connect^TM^ Real‐Time PCR Detection System (Bio‐Rad) and SYBR Green (TaKaRa). The thermal cycling conditions were as follows: initial denaturation at 95°C for 30 seconds, followed by 40 cycles at 95°C for 10 seconds and 60°C for 30 seconds. The mRNA levels were normalized to those of β‐actin RNA. The sequences of the primers used are as follows: Poldip2, forward 5′‐GAGGGGTCGTCCTGTTTCC‐3′ and reverse 5′‐GGGCATCAATCAGCACTTGG‐3′; β‐actin, forward 5′‐CAGCCTTCCTTCTTGGGTA‐3′ and reverse 5′‐TTTACGGATGTCAACGTCACAC‐3′.

### 2.13 Transmission electron microscopy (TEM)

Mice were anesthetized and transcardially perfused with ice‐cold 0.9% NaCl followed by fixing solution containing 4% paraformaldehyde and 1% glutaraldehyde. The brains were removed and cut into 1 mm × 1 mm × 1 mm blocks, followed by fixing in 2.5% glutaraldehyde overnight at 4°C and postfixing in 2% osmium tetroxide for 2 hours at 4°C. Next, the blocks were dehydrated in alcohol, passed through propylene oxide, embedded in Epon‐Araldite, and cut into ultrathin sections (100 nm). The prepared sections were stained with uranyl acetate and lead citrate and examined using TEM (H‐7500; Hitachi).

### 2.14 Evans blue (EB) permeability assay

For analysis of BBB permeability, mice were injected with 2% Evans blue (3 mL/kg) (Aladdin, China) intravenously.^34^ After three hours, mice were anesthetized with 3.5% chloral hydrate and transcardially perfused with 0.1 mol/L cold phosphate‐buffered saline (PBS, pH 7.4). Then, the brains were removed immediately and stored at − 80°C until use. The brains were homogenized in PBS, sonicated, and centrifuged for 30 minutes at 12 000 ***g*** and 4°C. The supernatant was collected and equal volume of trichloroacetic acid (TCA) was added, followed by incubation at 4°C for 24 hours. After centrifugation (12 000 ***g***, 4°C, 30 minutes), Evans blue absorbance was measured at 610 nm using a spectrophotometer (Thermo Fisher Scientific) and interpreted based on the standard curve.

### 2.15 Gelatin zymography

The process of protein extraction and total protein content detection was same as that of Western blotting.^35^ Protein samples (30 μg proteins/well) were resolved using 10% SDS‐polyacrylamide gel containing 0.1% gelatin at 100 V and 50 A for 2 hours. After that, the gel was washed twice with 2.5% Triton X‐100 and incubated in incubation buffer (50 mmol/L Tris‐HCl, 5mmol/L CaCl_2_, 1 μmol/L ZnCl_2_, 0.02% NaN_3_) for 20 hours at 37°C. Next, the gel was stained with Coomassie Brilliant Blue (Beyotime, P0017) and photographed. Band intensity was measured using Quantity One software (version 4.6.2).

### 2.16 Statistical analysis

Statistical analyses were performed using GraphPad Prism 6 (GraphPad software). Data are expressed as mean ± standard error of mean (SEM). KS normality test was used to assess the normality of data distribution. Statistical difference between groups was analyzed using one‐way analysis of variance (ANOVA) followed by multiple comparisons using Tukey's post hoc test. *P* values were two‐sided, and *P* < .05 was considered statistically significant.

## 3 RESULTS

### 3.1 Time course and spatial expression of endogenous Poldip2 in mouse BM model

**FIGURE 1:**
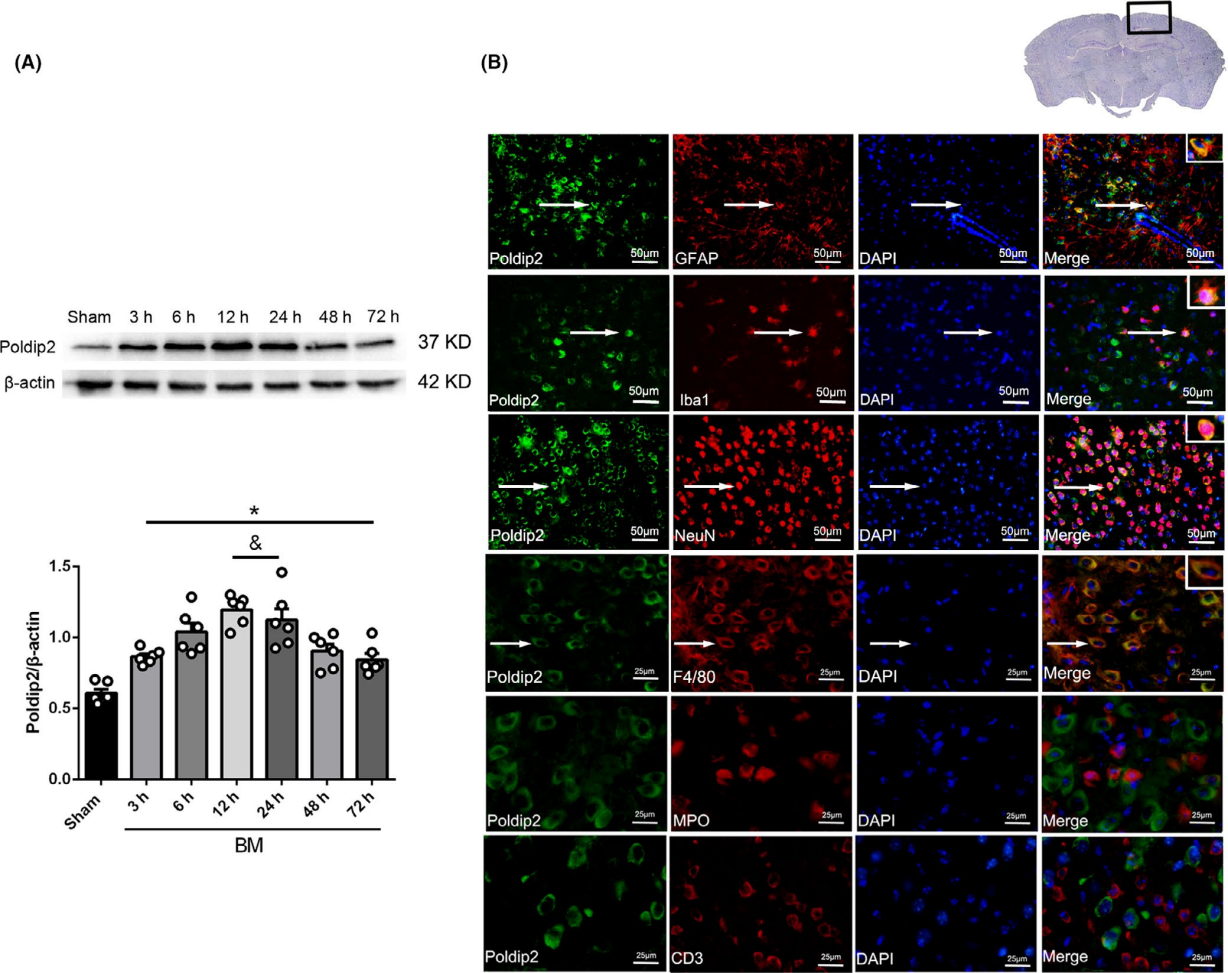
Endogenous expression of Poldip2 after BM induction. A, Representative Western blotting bands and quantitative analyses of endogenous Poldip2 after BM induction. Data are presented as mean ± SEM, n = 6 per group. *P < .05 vs. sham, ^&^ *P* < .05 vs. 3 h. B, Double immunofluorescence staining of Poldip2 with astrocytes (GFAP), neurons (NeuN), microglia/macrophages (Iba‐1 and F4/80), neutrophils (MPO), and T cell (CD3) at 12 hours after BM induction. The nuclei were stained by DAPI (blue). Arrows indicate the cells where Poldip2 is colocalized with GFAP, NeuN, Iba‐1, and F4/80. n = 3 per group. Top panel indicates the location of immunofluorescence staining (small black box). The three upper panels in immunofluorescence pictures, scale bar = 50 μm; the three lower panels in immunofluorescence pictures, scale bar = 25 μm

Endogenous Poldip2 expression increased significantly in a time‐dependent manner as compared to the sham control group (*P* < .05), reaching a peak value at 12 hours after BM induction (Figure 1A). Double immunofluorescence staining showed that Poldip2 colocalized with the astrocyte marker (GFAP), neuron marker (NeuN), and microglia/macrophage markers (Iba1 and F4/80), but not colocalized with neutrophils marker (MPO) and T cell marker (CD3) in the mouse brain at 12 hours after BM induction (Figure 1B).

### 3.2 In vivo knockdown of Poldip2 by Poldip2 siRNA

**FIGURE 2:**
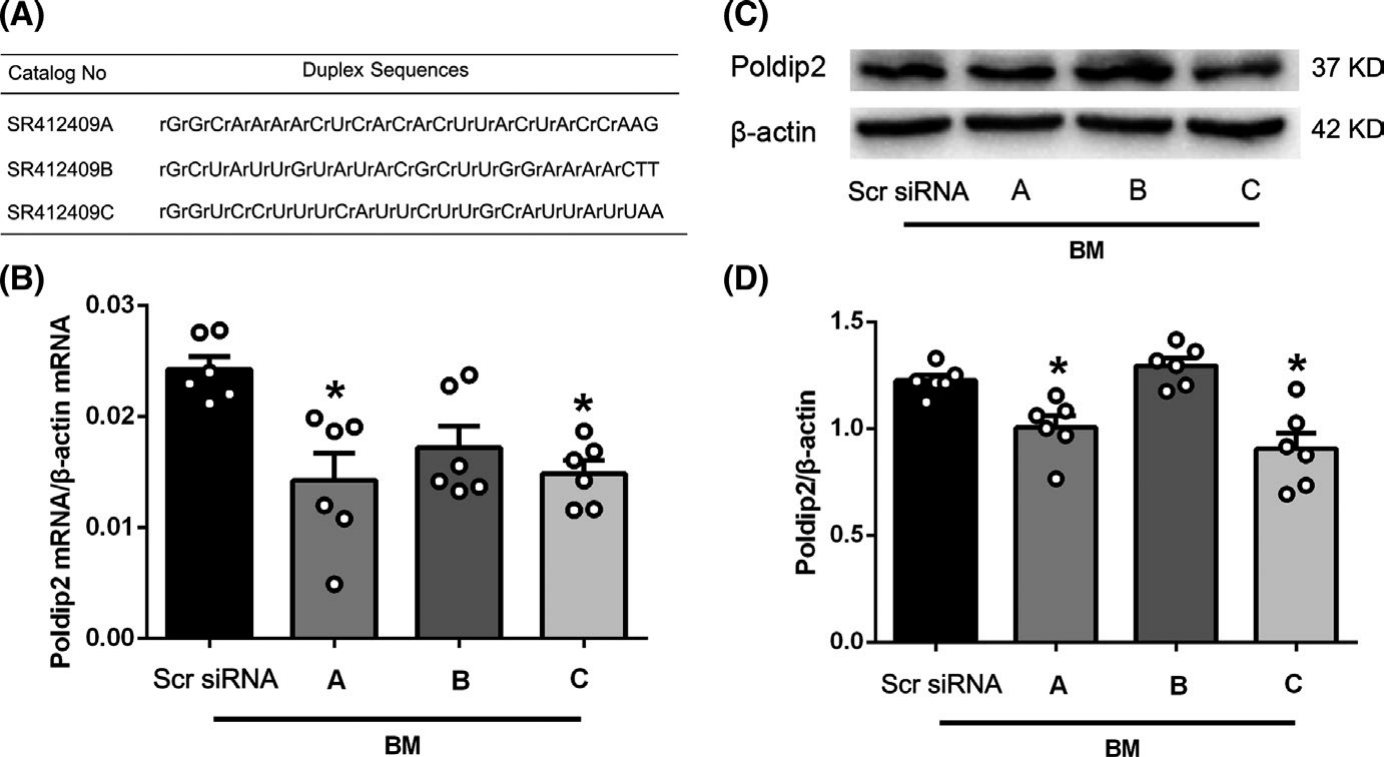
Effect of three candidate Poldip2 siRNA sequences on the expression level of Poldip2 mRNA and protein at 12 hours after BM induction. A, The sequences of three candidate Poldip2 siRNAs. B, Quantitative real‐time PCR results showing the effect of different Poldip2 siRNA sequences on Poldip2 mRNA expression. C‐D, Representative Western blotting bands and quantitative analyses of Poldip2 showing the effect of different Poldip2 siRNA sequences on Poldip2 protein expression. Data are presented as mean ± SEM, n = 6 per group. *P < .05 vs. scramble siRNA group

Three candidate Poldip2 siRNA sequences (A, B, and C; Figure 2A) were administered by i.c.v injection 48 hours before BM induction. Quantitative real‐time PCR analysis showed that Poldip2 mRNA expression decreased significantly in groups A and C as compared to that in the scramble group at 12 hours after BM induction (*P* < .05), while sequence B treatment had no significant effect on Poldip2 mRNA expression as compared to the scramble group (*P* > .05, Figure 2B). Western blotting revealed that Poldip2 protein expression decreased significantly in groups A and C (*P* < .05), but no significant difference was observed in group B (*P* > .05, Figure 2C,D) when compared to the scramble group. Considering that group C showed slightly lower Poldip2 expression than group A, though not significant, sequence C siRNA was selected for the further experiments.

### 3.3 Effect of endogenous Poldip2 knockdown on neurological status, brain edema, and BBB disruption in mouse BM model

**FIGURE 3:**
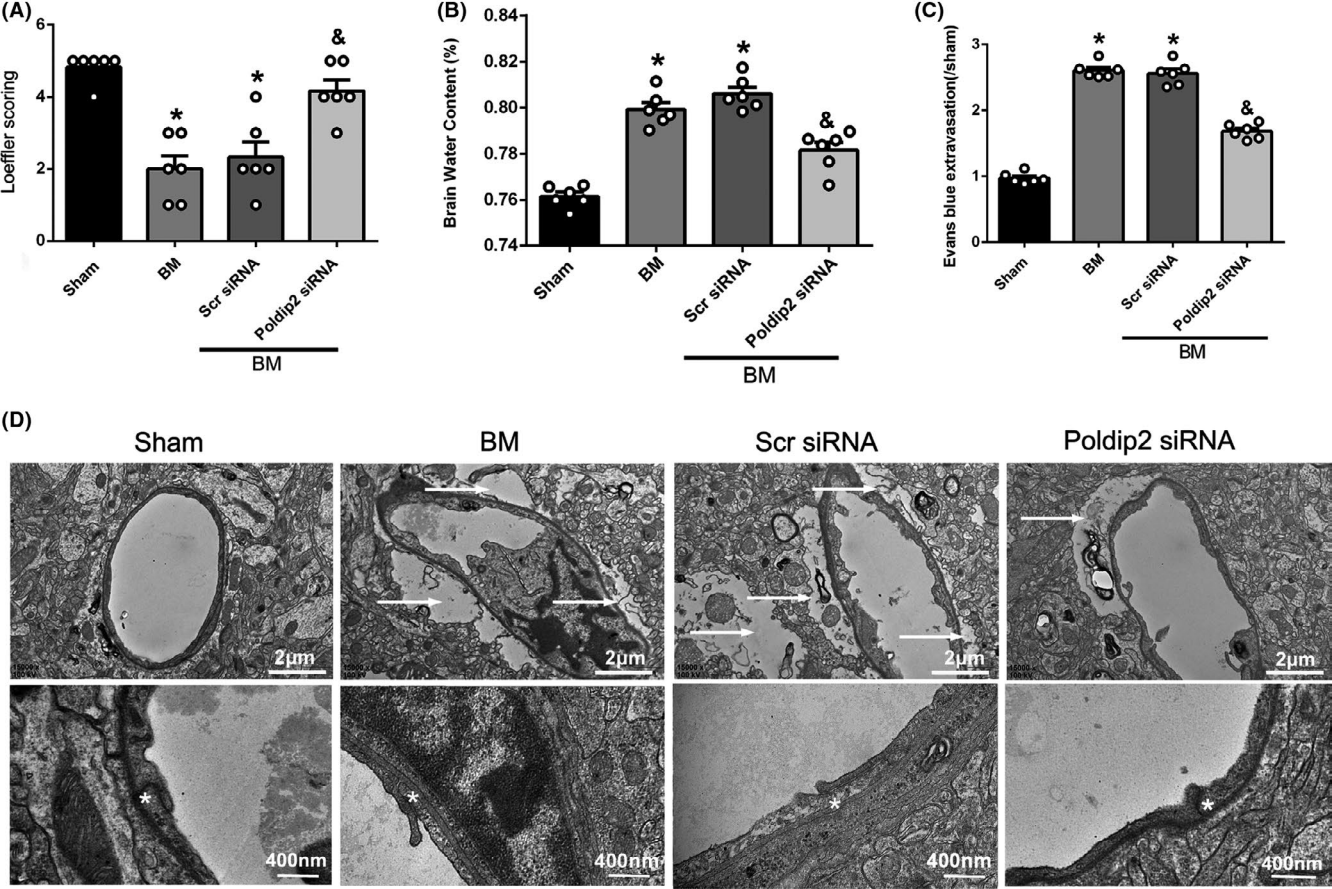
Effect of Poldip2 siRNA on neurological functions, brain water content, BBB permeability, and astrocyte swelling in mouse BM model at 12 hours after BM induction. A, Effect of Poldip2 siRNA on neurological function. Loeffler's neurological score was decreased significantly in BM group as compared to the sham group; Poldip2 siRNA improved the neurological score significantly as compared to the scramble siRNA group. Data are presented as mean ± SEM, n = 6 per group. *P < .05 vs. sham group, ^&^ *P* < .05 vs. scramble siRNA group. B, Effect of Poldip2 siRNA on brain water content. Brain water content increased in BM group as compared to the sham group and decreased in Poldip2 siRNA group as compared to the scramble siRNA group. Data are presented as mean ± SEM, n = 6 per group. *P < .05 vs. sham group, ^&^ *P* < .05 vs. scramble siRNA group. C, Effect of Poldip2 siRNA on BBB permeability. EB extravasation increased in BM group as compared to the sham group and decreased in Poldip2 siRNA group as compared to the scramble siRNA group. Data are presented as mean ± SEM, n = 6 per group. *P < .05 vs. sham group, ^&^ *P* < .05 vs. scramble siRNA group. D, Effect of Poldip2 siRNA on astrocyte swelling and tight junctions. The upper panel shows that the swelling area increased in the BM and scramble siRNA groups as compared to the sham group and decreased in the Poldip2 siRNA group as compared to the scramble siRNA group. The arrows indicate swollen astrocytes, scale bar = 2 μm. The lower panel shows that the tight junctions appeared as diffuse, disorganized structures in the BM and scramble siRNA groups, while in the Poldip2 siRNA group, the tight junctions were less disorganized. The asterisks (*) indicate the tight junctions, scale bar = 400 nm. n = 3 per group

In the BM group, at 12 h after BM induction, Loeffler's neurological score significantly decreased (*P* < .05, Figure 3A) and brain water content (*P* < .05, Figure 3B) and EB extravasation (*P* < .05, Figure 3C) increased as compared to the sham group. Whereas, in the Poldip2 siRNA group, Loeffler's neurological score significantly improved (*P* < .05, Figure 3A) and brain water content (*P* < .05, Figure 3B) and EB extravasation (*P* < .05, Figure 3C) decreased as compared to the scramble siRNA group. TEM demonstrated obvious astrocytic swelling and tight junctions appeared as diffuse, disorganized structures in BM (12 hours after BM induction) and scramble siRNA groups, while the Poldip2 siRNA group showed reduction in astrocytic swelling as well as less disorganized tight junctions (Figure 3D).

### 3.4 Effect of endogenous Poldip2 knockdown on AQP4 polarity loss in mouse BM model

**FIGURE 4:**
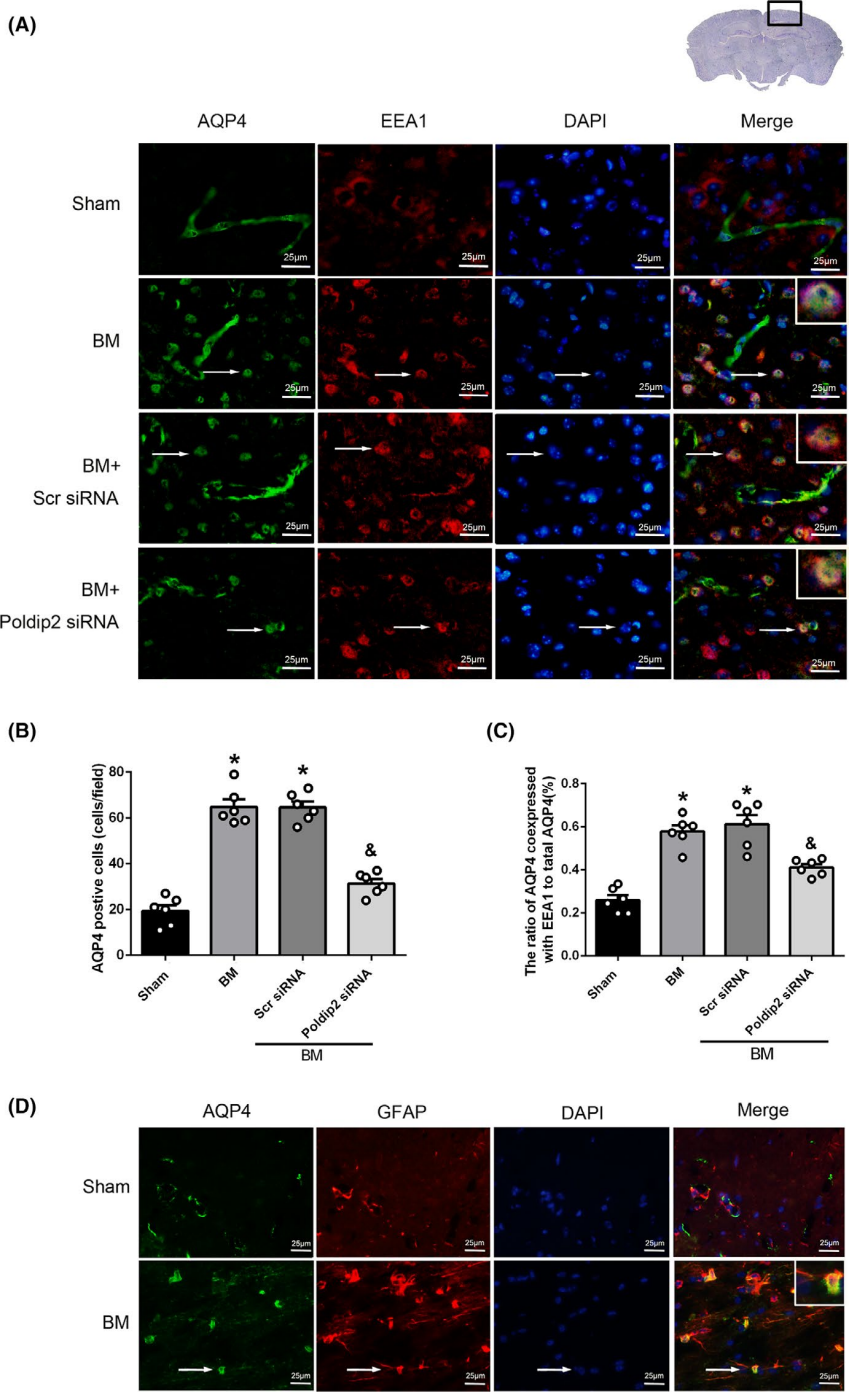
Effect of Poldip2 siRNA on polarized expression of AQP4. A, Double immunofluorescence images showing colocalization of AQP4 with EEA1 in sham, BM, BM + scramble siRNA, and BM + Poldip2 siRNA groups. The nuclei were stained by DAPI (blue). Top panel indicates the location of immunofluorescence staining (small black box). B‐C, Quantification of AQP4‐positive cells and ratio of AQP4 coexpressed with EEA1 to total AQP4 in different groups. Data are presented as mean ± SEM, n = 6 per group. *P < .05 vs. sham group, ^&^ *P* < .05 vs. scramble siRNA group. D, Double immunofluorescence of AQP4 with GFAP (marker of astrocytes) in the sham and BM groups. DAPI marked nuclei. Scale bar = 25 μm

Early endosome antigen 1 (EEA1) is a marker protein of early endosome. The colocalization of AQP4 with EEA1 indicates that the polarized localization of AQP4 at astrocyte membrane is lost and AQP4 is internalized into the cytoplasm containing early endosome. Double immunofluorescence staining of AQP4 and EEA1 showed that the number of AQP4‐positive cells and the ratio of AQP4 coexpressed with EEA1 to total AQP4 increased significantly in the cerebral cortex of BM group at 12 hours after BM induction as compared to the sham group (*P* < .05, Figure 4A‐C), but decreased in the Poldip2 siRNA group as compared to the scramble siRNA group (*P* < .05, Figure 4A‐C). Furthermore, double immunofluorescence staining of AQP4 and GFAP showed that AQP4 colocalized with astrocytic cytoplasm in the BM group at 12 hours after BM induction while AQP4 colocalized with astrocytic endfeet in the sham group (Figure 4D).

### 3.5 Effect of Rh‐Poldip2 and Poldip2 siRNA on the expression of MMPs, β‐DG, ZO‐1, occludin, claudin‐5, and GFAP

**FIGURE 5:**
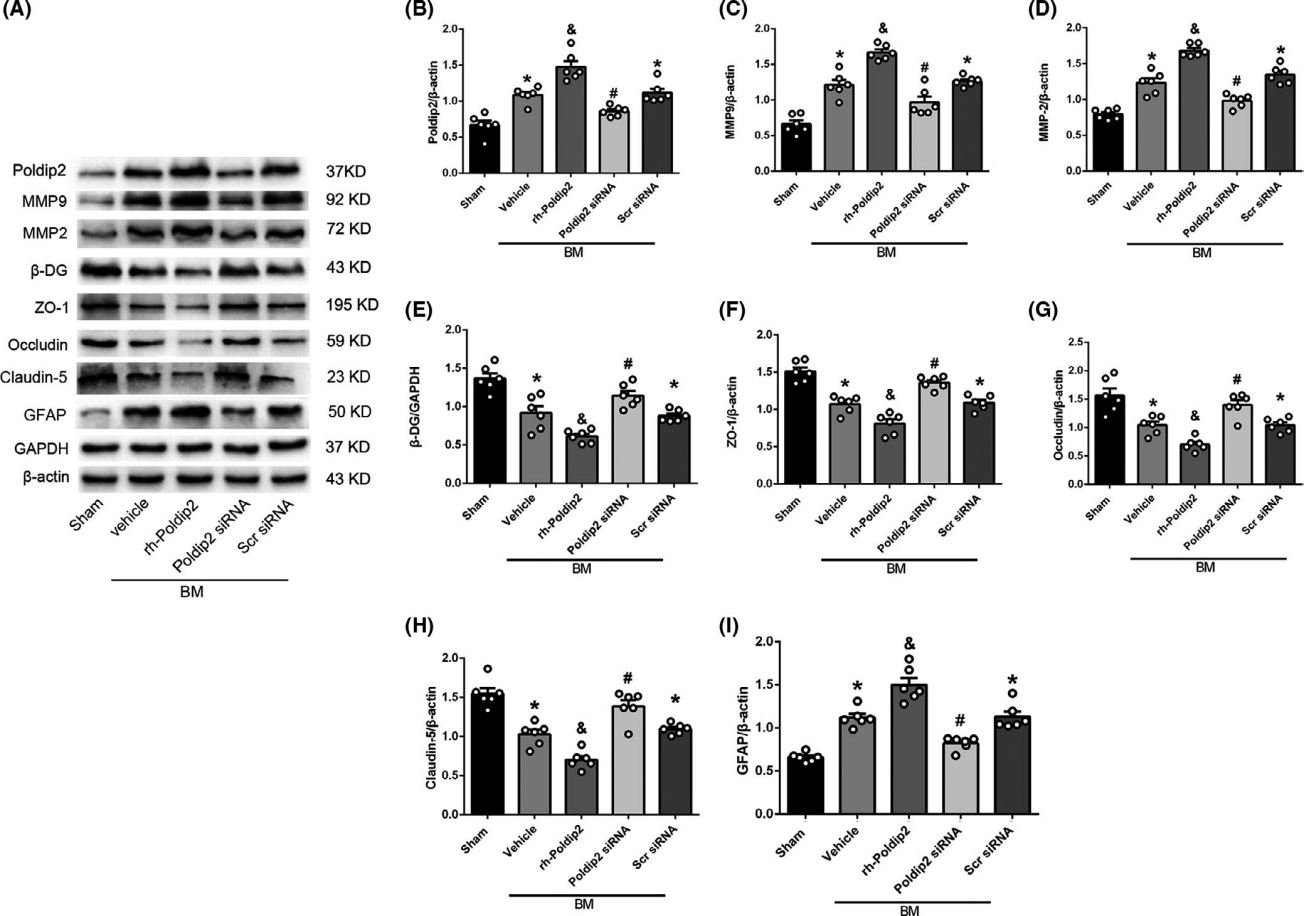
Effect of Poldip2 siRNA on the expression level of downstream proteins and GFAP at 12 hours after BM induction. A, Representative Western blotting bands of Poldip2, MMPs, β‐DG, ZO‐1, occludin, claudin‐5, and GFAP. B‐I, Quantitative analysis of Poldip2, MMPs, β‐DG, ZO‐1, occludin, claudin‐5, and GFAP expression levels. Data are presented as mean ± SEM, n = 6 per group. *P < .05 vs. sham group; ^&^ *P* < .05 vs. vehicle group; ^#^ *P* < .05 vs. scramble siRNA group

The expression levels of MMP2 and MMP9 were determined, as they are the most extensively studied MMPs in the brain. ZO‐1, occludin, and claudin‐5 expression levels were determined as they are crucial in maintaining the stability of tight junctions and BBB permeability. β‐DG expression level was investigated because it is a substrate for one of the MMPs and is crucial for AQP4 anchoring on the astrocyte membrane. GFAP expression level was investigated as it is related to AQP4 polarity. Western blot results showed that the expression levels of Poldip2, MMP9, MMP2, and GFAP increased and those of β‐DG, ZO‐1, occludin, and claudin‐5 decreased in the vehicle group as compared to the sham group (*P* < .05). Rh‐Poldip2 administration upregulated Poldip2, MMP9, MMP2, and GFAP expression, and downregulated β‐DG, ZO‐1, occludin, and claudin‐5 expression as compared to the vehicle group (*P* < .05). The scramble siRNA group showed increased expression of Poldip2, MMP9, MMP2, and GFAP, and decreased expression of β‐DG, ZO‐1, occludin, and claudin‐5 as compared to the sham group (*P* < .05). Poldip2 siRNA downregulated Poldip2, MMP9, MMP2, and GFAP expression, and upregulated β‐DG, ZO‐1, occludin, and claudin‐5 expression as compared to the scramble siRNA group (*P* < .05, Figure 5).

**FIGURE 6:**
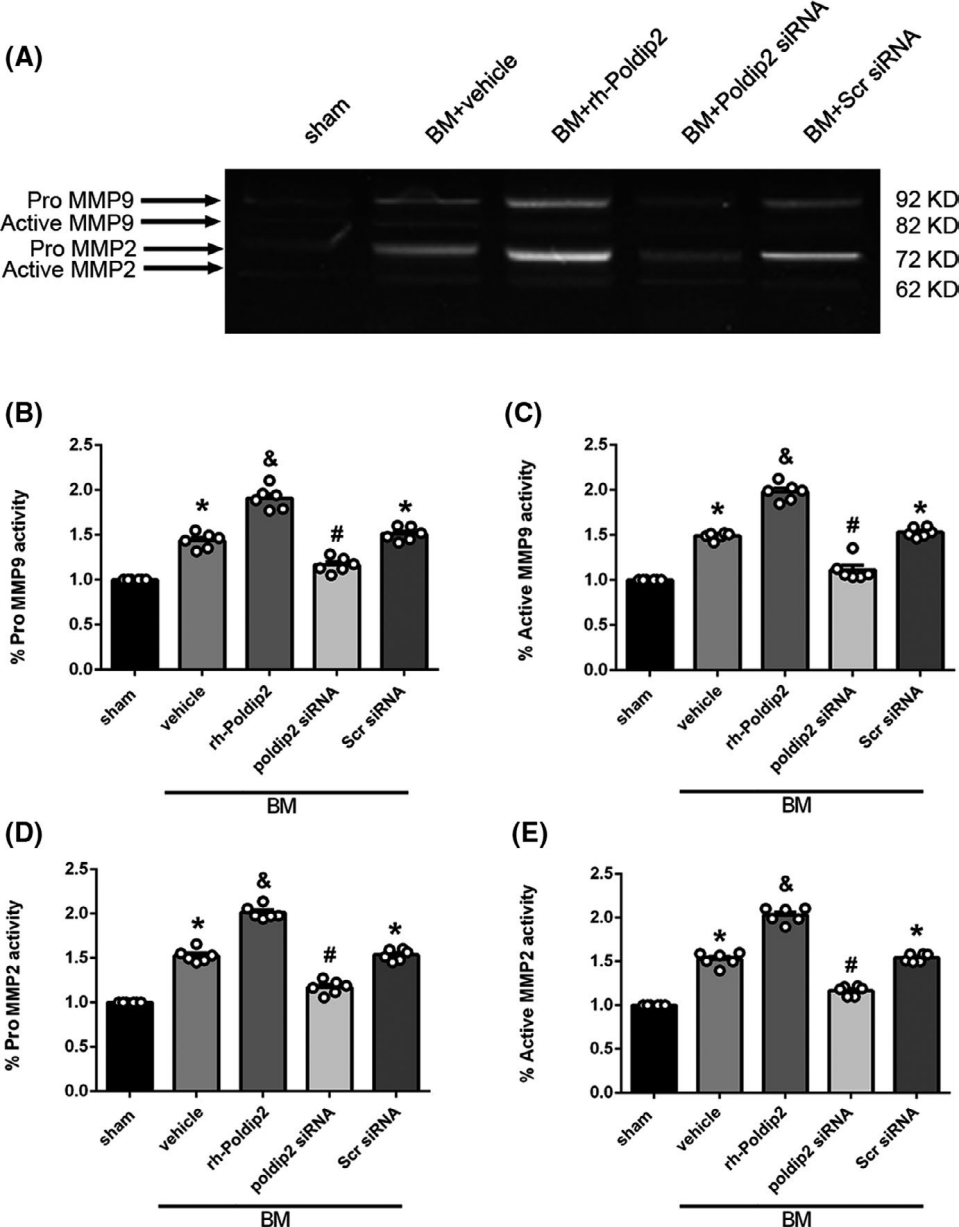
Effect of Poldip2 siRNA on the activity of MMP9 and MMP2 at 12 h after BM induction. A, Representative photographs of pro‐MMP2, active MMP2, pro‐MMP9, and active MMP9. B‐E, Quantitative analysis of pro‐MMP2, active MMP2, pro‐MMP9, and active MMP9 activity levels. Data are presented as mean ± SEM, n = 6 per group. *P < .05 vs. sham group; ^&^ *P* < .05 vs. vehicle group; ^#^ *P* < .05 vs. scramble siRNA group

Gelatin zymography showed that the activity of MMP9 and MMP2 in vehicle group was higher than that in the sham group (*P* < .05). Rh‐Poldip2 administration further upregulated the activity of MMP9 and MMP2 as compared to the vehicle group (*P* < .05). The activity of MMP9 and MMP2 in the scramble siRNA group was higher than that in the sham group (*P* < .05). Poldip2 siRNA downregulated the activity of MMP9 and MMP2 as compared to the scramble siRNA group (*P* < .05, Figure 6).

### 3.6 Effect of MMPs inhibitor on neurological deficits, AQP4 polarization, brain edema, and BBB permeability after BM induction

**FIGURE 7:**
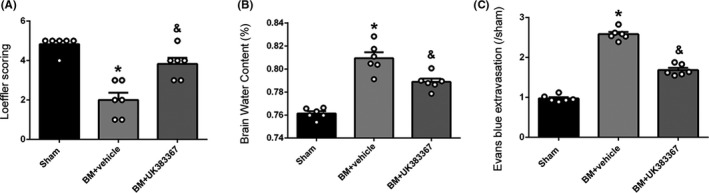
Effect of the MMPs inhibitor UK383367 on neurological functions, brain water content, and BBB permeability at 12 hours after BM induction. A, Effect of UK383367 on neurological function. Loeffler's neurological score decreased significantly in the vehicle group as compared to the sham group and increased significantly in the UK383367 group as compared to the vehicle group. B, Effect of UK383367 on brain water content. The brain water content increased in the vehicle group as compared to the sham group and decreased significantly in the UK383367 group as compared to the vehicle group. C, Effect of UK383367 on BBB permeability. EB extravasation increased in the vehicle group as compared to the sham group and decreased significantly in the UK383367 group as compared to the vehicle group. Data are presented as mean ± SEM, n = 6 per group. *P < .05 vs. sham group, ^&^ *P* < .05 vs. vehicle group

In the vehicle group at 12 hours after BM induction, Loeffler's neurological score significantly decreased (*P* < .05, Figure 7A) and brain water content (*P* < .05, Figure 7B) and EB extravasation (*P* < .05, Figure 7C) increased as compared to the sham group. Whereas, in the UK383367 group, Loeffler's neurological score increased (*P* < .05, Figure 7A) and brain water content (*P* < .05, Figure 7B) and EB extravasation (*P* < .05, Figure 7C) decreased as compared to the vehicle group.

**FIGURE 8:**
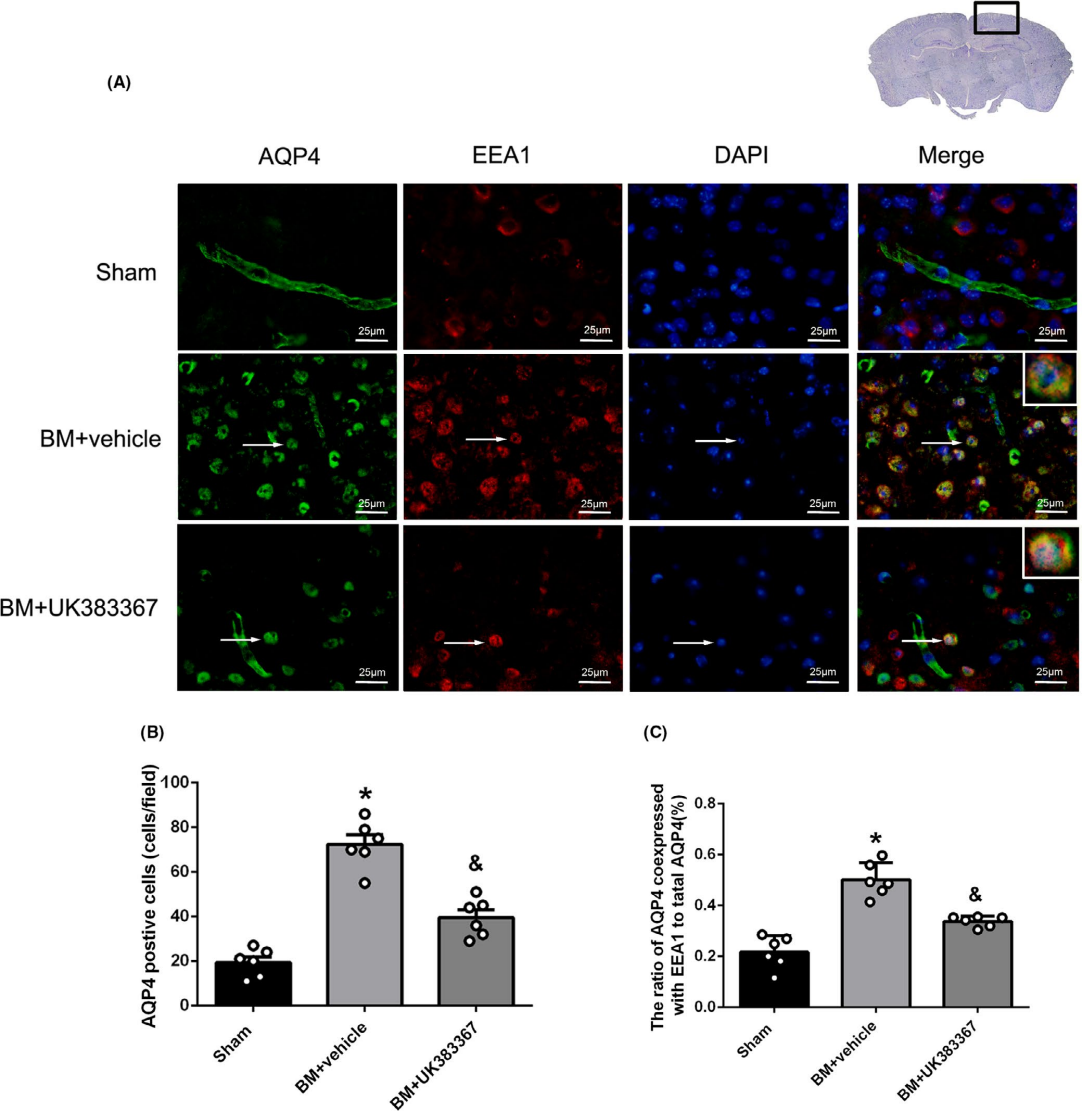
Effect of the MMPs inhibitor UK383367 on AQP4 polarized expression. A, Double immunofluorescence images showing colocalization of AQP4 with EEA1 in the sham, BM + vehicle, and BM + UK383367 groups. The nuclei were stained by DAPI (blue). Top panel indicates the location of immunofluorescence staining (small black box). Scale bar = 25 μm. B‐C, Quantification of AQP4‐positive cells and ratio of AQP4 coexpressed with EEA1 to total AQP4 in different groups. Data are presented as mean ± SEM, n = 6 per group. *P < .05 vs. sham group, ^&^ *P* < .05 vs. vehicle group

Double immunofluorescence staining of AQP4 and EEA1 showed that the number of AQP4‐positive cells and the ratio of AQP4 coexpressed with EEA1 to total AQP4 increased significantly in the vehicle group at 12 hours after BM induction as compared to the sham group (*P* < .05, Figure 8) but decreased in the UK383367 group as compared to the vehicle group (*P* < .05, Figure 8).

**FIGURE 9:**
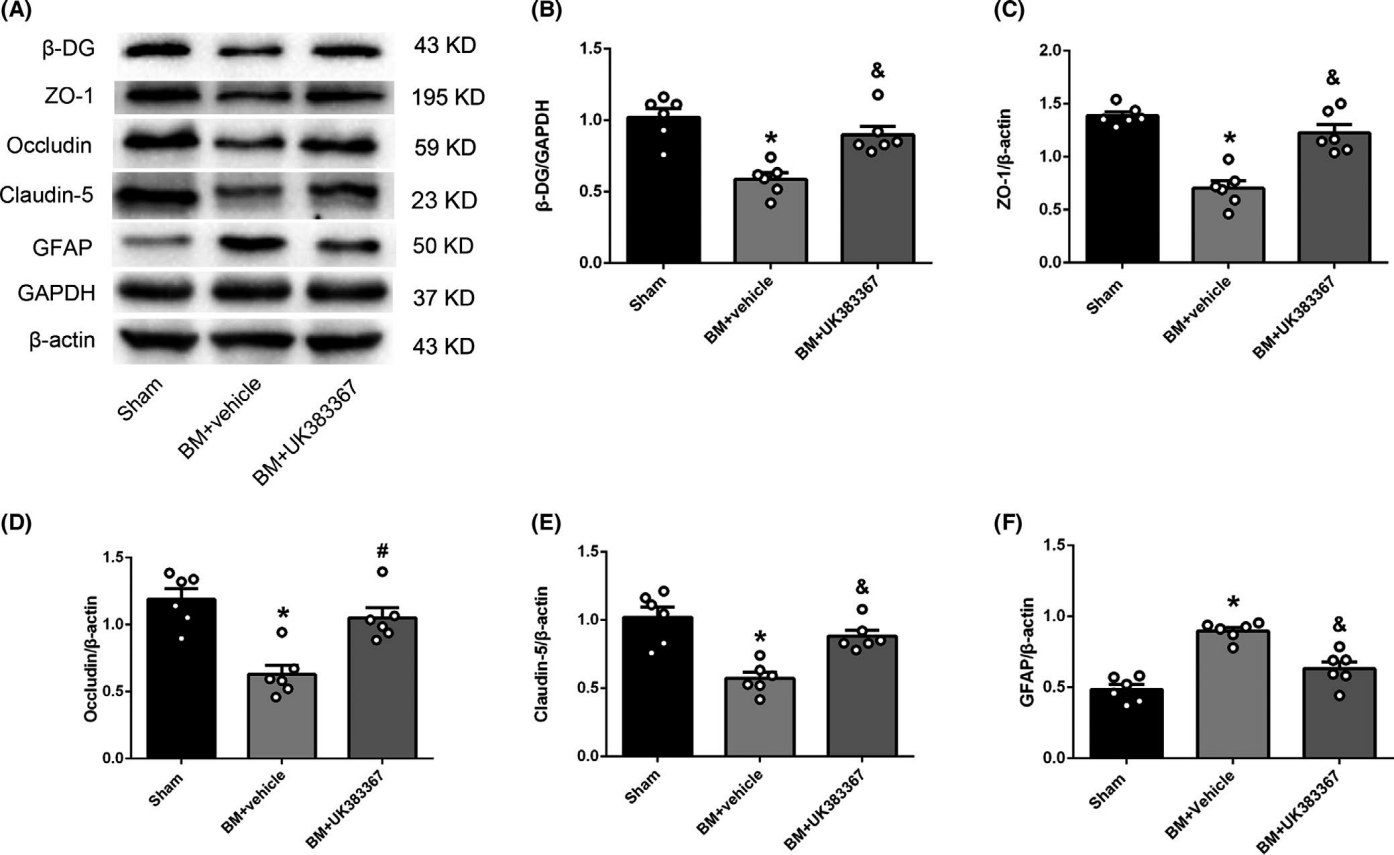
Effect of the MMPs inhibitor UK383367 on the expression level of downstream proteins and GFAP at 12 hours after BM induction. A, Representative Western blotting bands of β‐DG, ZO‐1, occludin, claudin‐5, and GFAP. B‐F, Quantitative analysis of β‐DG, ZO‐1, occludin, claudin‐5, and GFAP expression levels. Data are presented as mean ± SEM, n = 6 per group. *P < .05 vs. sham group, ^&^ *P* < .05 vs. vehicle group

Western blotting showed that the expression levels of β‐DG, ZO‐1, occludin, and claudin‐5 decreased and GFAP expression level increased in the BM induction group as compared to the sham group (*P* < .05), while the expression levels of β‐DG, ZO‐1, occludin, and claudin‐5 increased and GFAP expression level decreased in the UK383367 group as compared to the vehicle group (*P* < .05, Figure 9).

## 4 DISCUSSION

Poldip2 is expressed extensively in the central nervous system and peripheral tissues, including brain, thyroid, heart, blood vessels, lungs, and kidneys.^24^, ^36^, ^37^, ^38^, ^39^, ^40^ Many studies have reported the role of Poldip2 in peripheral tissues,^23^, ^36^, ^37^, ^38^, ^39^, ^40^ but its role in the brain has not been well studied and is limited to very few investigations.^23^, ^24^, ^41^ In the present study, we observed that Poldip2 is expressed extensively in various types of cells in the mouse brain, suggesting the need to investigate the physiological and pathological functions of Poldip2 in the central nervous system. Endogenous Poldip2 expression was significantly increased in the brain of mouse BM model, indicating that Poldip2 plays a role in the pathogenesis of BM.

Knowing that increased Poldip2 accounted for the breakdown of the BBB following ischemic stroke ^24^ and considering the astrocytic expression of Poldip2 in our study, we investigated the effect of Poldip2 upregulation on BBB integrity and brain edema formation in the BM model. We observed that exogenous Poldip2 delivery downregulated the tight junction proteins ZO‐1, occludin, and claudin‐5, whereas Poldip2 siRNA upregulated these proteins and reduced EB extravasation. These results indicate that Poldip2 mediates the breakdown of BBB in mouse BM model. Meanwhile, Poldip2 siRNA reduced the brain water content and improved the neurobehavioral score in BM mouse, which further proved that Poldip2 inhibition is beneficial for alleviating neurological impairment in BM.

Accumulating evidence reveals that AQP4 facilitates the elimination of edema fluid in vasogenic edema,^42^, ^43^ one of the main types of brain edema, characterized by BBB disruption. AQP4 polarization on the astrocyte endfeet is the structural basis for its water transport function; therefore, loss of AQP4 polarity will disrupt BBB integrity and mediate brain edema formation.

AQP4 expression level was greatly increased in mouse BM model, which is consistent with other studies.^44^, ^45^ For water transportation function, AQP4 expression at brain‐blood interfaces is essential, suggesting that a change in the location of AQP4 expression is important rather than a change in AQP4 expression level. The ratio of AQP4 coexpressed with EEA1 to total AQP4 increased in mouse BM model and decreased after Poldip2 siRNA injection, revealing that AQP4 polarization was lost in BM and Poldip2 inhibition could reverse the loss of AQP4 polarity in BM brain. However, the underlying mechanism of AQP4 polarity loss mediated by Poldip2 remains to be explored.

It has been demonstrated that Poldip2 is responsible for the upregulation of MMPs activity in hindlimb ischemia‐induced mice and stroke animal model,^24^, ^25^ but very little is known about the molecular mechanism of Poldip2 in regulating MMPs. Based on certain clues from existing literature, we speculate that Poldip2 might regulate the expression and activity of MMPs through nicotinamide adenine dinucleotide phosphate (NAPDH) oxidase 4 (NOX4) or nuclear factor‐κB (NF‐κB) pathway. ①NOX4 pathway. Poldip2 was identified as a unique positive regulator of NOX4 through interaction with p22^phox^ in various tissue and cells, such as vascular smooth muscle cells, renal pelvis of rats, and thyroid glands.^37^, ^38^, ^46^ NOX4‐mediated reactive oxygen species (ROS) production is related to the activation of Forkhead box O (FoxO) transcription factors,^47^, ^48^ which are involved in MMPs regulation. For example, MMP2, MMP3, and MMP9 are direct or indirect target genes of FoxO3a in endothelial as well as cancer cells,^48^, ^49^, ^50^ and FoxO4 upregulated MMP9 expression in smooth muscle cells stimulated by tumor necrosis factor‐α (TNF‐α) by an indirect mechanism.^51^ Therefore, Poldip2/Nox4/FoxO/MMPs is one possible pathway for MMPs regulation by Poldip2. ②NF‐κB pathway. NF‐κB is a family of transcription factors comprising five different proteins, named p65, RelB, c‐Rel, p50, and p52. It was demonstrated that Poldip2 regulated NF‐κB activity through the p65 subunit in LPS‐induced BBB disruption animal model.^52^ Meanwhile, NF‐κB is extensively reported to be involved in the regulation of MMPs in vivo and in vitro.^53^, ^54^, ^55^, ^56^ For example, NF‐κB complexes interact with the MMP9 promoter directly in human coronary artery smooth muscle cells. MMP9 transcription was significantly attenuated by p65 knockdown but induced by ectopic expression of wild‐type p50 or p65.^57^ Considering the relationship of Poldip2 with NF‐κB, and NF‐κB with MMPs, Poldip2/NF‐κB/MMPs is another possible pathway for MMP regulation by Poldip2. Our study demonstrated that the expression and activity of both MMP2 and MMP9 in BM animal model are upregulated by exogenous Poldip2 and downregulated by Poldip2 knockdown, corroborating the results of previous studies in hindlimb ischemia‐induced mice and stroke animal model. However, further investigation is required to understand the detailed mechanism of MMP regulation by Poldip2 in BM model.

The localization of AQP4 anchoring at the perivascular endfeet of glial cells is dependent on the function of DGC components, as observed by the dramatic reduction of AQP4 immunoreactivity in perivascular astrocyte membranes in the brains of dystrophin‐deficient mdx mice and α‐syntrophin‐deficient (α‐syn−/−) mouse.^13^, ^58^, ^59^, ^60^ Among the components of DGC, DG plays a central role in anchoring AQP4 at the proper membrane domain.^61^ The two isoforms of DG are α‐DG and β‐DG; α‐DG binds to ECM components such as agrin and laminin, whereas β‐DG is a membrane‐spanning protein connecting α‐DG to α‐syntrophin, another component of DGC, which further interacts with cytoskeleton and AQP4.^17^, ^62^, ^63^ At the astrocyte endfeet, DG is recruited as clusters and they anchor AQP4 through their link with laminin presented in the perivascular basal lamina. Tham et al demonstrated that the interaction of DG with dynamin could regulate the trafficking of AQP4 between astrocyte membrane and the recycling endosomes in cytoplasm.^61^, ^62^, ^63^ DG preferentially associates with the “inactive” GDP‐bound form of dynamin and inhibits the endocytosis of AQP4.^62^, ^64^ Cleavage of β‐DG will lead to the disruption of AQP4’s asymmetric enrichment at the perivascular astrocyte endfeet and promote AQP4 internalization to endosomes.

Numerous studies have reported that β‐DG is one of the substrates of MMPs.^21^, ^22^ For example, MMPs mediated the cleavage of β‐DG in myelin sheath in autoimmune neuritis.^22^ β‐DG could be cleaved by MMPs, especially MMP9, in Duchenne muscular dystrophy.^21^ Since Poldip2 could activate MMPs and MMPs could cleave β‐DG,^21^, ^22^, ^24^, ^25^ we explored the role of MMPs in Poldip2‐mediated β‐DG degradation and AQP4 polarity loss. We observed that the MMP inhibitor UK383367 downregulated β‐DG expression and increased the ratio of AQP4 coexpressed with EEA1 to total AQP4, indicating that UK383367 alleviated the loss of AQP4 polarity. Furthermore, UK383367 increased the expression of tight junction proteins, reduced EB extravasation, alleviated brain edema, and improved neurobehavioral test scores. These results suggest that MMPs are at least partially involved in Poldip2‐mediated AQP4 polarity loss and play important roles in BBB disruption and brain edema formation in mouse BM model.

In conclusion, our study showed for the first time that endogenous Poldip2 expression was increased in the brain of mouse BM model, and Poldip2 inhibition could alleviate brain edema and BBB disruption by reversing AQP4 polarity loss via MMPs/β‐DG pathway. The mechanism of AQP4 polarity loss being induced by Poldip2 in mouse bacterial meningitis model is summarized in graphical abstract.

Moreover, Poldip2 was expressed not only in astrocytes but also in microglia and neurons, which suggests that Poldip2 may play multiple roles in inflammation, neuronal apoptosis, and so on. However, our study focused only on the role of Poldip2 in BBB integrity and brain edema in mouse BM model; its effects on inflammation and neuronal apoptosis need to be elucidated in further studies.

## CONFLICTS OF INTEREST

The authors declare that they have no conflicts of interest.

## SUPPLEMENTARY DATA

The original images of western blots can be found at Appendix S1.

## Supporting information

Appendix S1

## References

[1] Ouchenir L , Renaud C , Khan S , et al. The epidemiology, management, and outcomes of bacterial meningitis in infants. Pediatrics. 2017;140(1):e20170476. 28600447 10.1542/peds.2017-0476

[2] Skoff TH , Farley MM , Petit S , et al. Increasing burden of invasive group B streptococcal disease in nonpregnant adults, 1990–2007. Clin Infect Dis. 2009;49(1):85‐92. 19480572 10.1086/599369

[3] Kim KS . Investigating bacterial penetration of the blood‐brain barrier for the pathogenesis, prevention, and therapy of bacterial meningitis. ACS Infect Dis. 2020;6(1):34–42. 31805229 10.1021/acsinfecdis.9b00319

[4] Pschibul A , Janzarik WG , Franck P , Hufnagel M , Beck C , Korinthenberg R . Cystic encephalomalacia following vasculopathy and vasospasm of proximal intracranial arteries due to pneumococcal meningitis in a infant. Neuropediatrics. 2018;49(3):213‐216. 29523004 10.1055/s-0038-1635075

[5] Stokum JA , Gerzanich V , Simard JM . Molecular pathophysiology of cerebral edema. J Cereb Blood Flow Metab. 2016;36(3):513‐538. 26661240 10.1177/0271678X15617172 PMC4776312

[6] Tasker RC , Acerini CL . Cerebral edema in children with diabetic ketoacidosis: vasogenic rather than cellular? Pediatr. Diabetes. 2014;15(4):261‐270. 24866062 10.1111/pedi.12153

[7] Assentoft M , Larsen BR , MacAulay N . Regulation and function of AQP4 in the central nervous system. Neurochem Res. 2015;40(12):2615‐2627. 25630715 10.1007/s11064-015-1519-z

[8] Hoddevik EH , Khan FH , Rahmani S , Ottersen OP , Boldt HB , Amiry‐Moghaddam M . Factors determining the density of AQP4 water channel molecules at the brain‐blood interface. Brain Struct Funct. 2017;222(4):1753‐1766. 27629271 10.1007/s00429-016-1305-y PMC5406442

[9] Xu M , Xiao M , Li S , Yang B . Aquaporins in nervous system. Adv Exp. Med Biol. 2017;969:81‐103. 28258567 10.1007/978-94-024-1057-0_5

[10] Park H , Choi SH , Kong MJ , Kang TC . Dysfunction of 67‐kDa laminin receptor disrupts BBB integrity via impaired dystrophin/AQP4 complex and p38 MAPK/VEGF activation following status epilepticus. Front Cell Neurosci. 2019;13:236. 31178701 10.3389/fncel.2019.00236 PMC6542995

[11] Zhao F , Deng J , Xu X , et al. Aquaporin‐4 deletion ameliorates hypoglycemia‐induced BBB permeability by inhibiting inflammatory responses. J Neuroinflammation. 2018;15(1):157. 29793504 10.1186/s12974-018-1203-8 PMC5968550

[12] Guo J , Mi X , Zhan R , Li M , Wei L , Sun J . Aquaporin 4 silencing aggravates hydrocephalus induced by injection of autologous blood in rats. Med Sci Monit. 2018;24:4204‐4212. 29921834 10.12659/MSM.906936 PMC6042309

[13] Amiry‐Moghaddam M , Frydenlund DS , Ottersen OP . Anchoring of aquaporin‐4 in brain: molecular mechanisms and implications for the physiology and pathophysiology of water transport. Neuroscience. 2004;129(4):999‐1010. 15561415 10.1016/j.neuroscience.2004.08.049

[14] Gan SW , Ran JH , Chen H , et al. Lysosomal degradation of retinal glial AQP4 following its internalization induced by acute ocular hypertension. Neurosci Lett. 2012;516(1):135‐140. 22490881 10.1016/j.neulet.2012.03.075

[15] Huang J , Lu WT , Sun SQ , et al. Upregulation and lysosomal degradation of AQP4 in rat brains with bacterial meningitis. Neurosci Lett. 2014;566:156‐161. 24602980 10.1016/j.neulet.2014.02.054

[16] Huang J , Sun SQ , Lu WT , et al. The internalization and lysosomal degradation of brain AQP4 after ischemic injury. Brain Res. 2013;1539:61‐72. 24070677 10.1016/j.brainres.2013.09.022

[17] Qiu GP , Xu J , Zhuo F , et al. Loss of AQP4 polarized localization with loss of beta‐dystroglycan immunoreactivity may induce brain edema following intracerebral hemorrhage. Neurosci Lett. 2015;588:42‐48. 25545558 10.1016/j.neulet.2014.12.053

[18] Pocsai K , Bagyura Z , Kalman M . Components of the basal lamina and dystrophin‐dystroglycan complex in the neurointermediate lobe of rat pituitary gland: different localizations of beta‐dystroglycan, dystrobrevins, alpha1‐syntrophin, and aquaporin‐4. J Histochem Cytochem. 2010;58(5):463‐479. 20124096 10.1369/jhc.2010.954768 PMC2857818

[19] Sato J , Horibe S , Kawauchi S , Sasaki N , Hirata KI , Rikitake Y . Involvement of aquaporin‐4 in laminin‐enhanced process formation of mouse astrocytes in 2D culture: roles of dystroglycan and alpha‐syntrophin in aquaporin‐4 expression. J Neurochem. 2018;147(4):495‐513. 29981530 10.1111/jnc.14548

[20] Waite A , Brown SC , Blake DJ . The dystrophin‐glycoprotein complex in brain development and disease. Trends Neurosci. 2012;35(8):487‐496. 22626542 10.1016/j.tins.2012.04.004

[21] Ogura Y , Tajrishi MM , Sato S , Hindi SM , Kumar A . Therapeutic potential of matrix metalloproteinases in Duchenne muscular dystrophy. Front Cell Dev Biol. 2014;2:11. 25364719 10.3389/fcell.2014.00011 PMC4207008

[22] Zhao XL , Li GZ , Sun B , et al. MMP‐mediated cleavage of beta‐dystroglycan in myelin sheath is involved in autoimmune neuritis. Biochem Biophys Res Commun. 2010;392(4):551‐556. 20097170 10.1016/j.bbrc.2010.01.062

[23] Hernandes MS , Lassegue B , Griendling KK . Polymerase delta‐interacting Protein 2: a multifunctional protein. J Cardiovasc Pharmacol. 2017;69(6):335‐342. 28574953 10.1097/FJC.0000000000000465 PMC5556945

[24] Hernandes MS , Lassegue B , Hilenski LL , et al. Polymerase delta‐interacting protein 2 deficiency protects against blood‐brain barrier permeability in the ischemic brain. J Neuroinflammation. 2018;15(1):45. 29452577 10.1186/s12974-017-1032-1 PMC5816395

[25] Amanso AM , Lassegue B , Joseph G , et al. Polymerase delta‐interacting protein 2 promotes postischemic neovascularization of the mouse hindlimb. Arterioscler Thromb Vasc Biol. 2014;34(7):1548‐1555. 24855063 10.1161/ATVBAHA.114.303873 PMC4146458

[26] Grandgirard D , Steiner O , Tauber MG , Leib SL . An infant mouse model of brain damage in pneumococcal meningitis. Acta Neuropathol. 2007;114(6):609‐617. 17938941 10.1007/s00401-007-0304-8

[27] Zhu Q , Enkhjargal B , Huang L , et al. Aggf1 attenuates neuroinflammation and BBB disruption via PI3K/Akt/NF‐kappaB pathway after subarachnoid hemorrhage in rats. J Neuroinflammation. 2018;15(1):178. 29885663 10.1186/s12974-018-1211-8 PMC5994242

[28] Nowrangi DS , McBride D , Manaenko A , Dixon B , Tang J , Zhang JH . rhIGF‐1 reduces the permeability of the blood‐brain barrier following intracerebral hemorrhage in mice. Exp Neurol. 2019;312:72‐81. 30503192 10.1016/j.expneurol.2018.11.009

[29] Allan GA , Gedge JI , Nedderman AN , Roffey SJ , Small HF , Webster R . Pharmacokinetics and metabolism of UK‐383,367 in rats and dogs: a rationale for long‐lived plasma radioactivity. Xenobiotica. 2006;36(5):399‐418. 16854779 10.1080/00498250600618177

[30] Hu N , Long H , Zhao M , Yin H , Lu Q . Aberrant expression pattern of histone acetylation modifiers and mitigation of lupus by SIRT1‐siRNA in MRL/lpr mice. Scand J Rheumatol. 2009;38(6):464‐471. 19922023 10.3109/03009740902895750

[31] Loeffler JM , Ringer R , Hablutzel M , Tauber MG , Leib SL . The free radical scavenger alpha‐phenyl‐tert‐butyl nitrone aggravates hippocampal apoptosis and learning deficits in experimental pneumococcal meningitis. J Infect Dis. 2001;183(2):247‐252. 11110643 10.1086/317921

[32] Zhang Y , Ding Y , Lu T , et al. Biliverdin reductase‐A attenuated GMH‐induced inflammatory response in the spleen by inhibiting toll‐like receptor‐4 through eNOS/NO pathway. J Neuroinflammation. 2018;15(1):118. 29678206 10.1186/s12974-018-1155-z PMC5910618

[33] Xiong J , Quan J , Qin C , Wang X , Dong Q , Zhang B . Dexmedetomidine exerts brain‐protective effects under cardiopulmonary bypass through inhibiting the Janus kinase 2/Signal transducers and activators of transcription 3 pathway. J Interferon Cytokine Res. 2020;40(2):116–124. 31834821 10.1089/jir.2019.0110

[34] Xie Z , Enkhjargal B , Reis C , et al. Netrin‐1 preserves blood‐brain barrier integrity through deleted in colorectal cancer/focal adhesion kinase/RhoA signaling pathway following subarachnoid hemorrhage in rats. J Am Heart Assoc. 2017;6(5):e005198. 28526701 10.1161/JAHA.116.005198 PMC5524080

[35] Chowdhury P , Dey P , De D , Ghosh U . Gamma ray induced in‐vitro cell migration via EGFR/ERK/Akt/p38 activation is prevented by olaparib pre‐treatment. Int J Radiat Biol. 2020;96(5):651–660. 31914341 10.1080/09553002.2020.1711461

[36] Chen YC , Kuo CC , Chian CF , et al. Knockdown of POLDIP2 suppresses tumor growth and invasion capacity and is linked to unfavorable transformation ability and metastatic feature in non‐small cell lung cancer. Exp Cell Res. 2018;368(1):42‐49. 29684384 10.1016/j.yexcr.2018.04.011

[37] Lin CS , Lee SH , Huang HS , Chen YS , Ma MC . H2O2 generated by NADPH oxidase 4 contributes to transient receptor potential vanilloid 1 channel‐mediated mechanosensation in the rat kidney. Am J Physiol Renal Physiol. 2015;309(4):F369‐F376. 26136558 10.1152/ajprenal.00462.2014

[38] Lyle AN , Deshpande NN , Taniyama Y , et al. Poldip2, a novel regulator of Nox4 and cytoskeletal integrity in vascular smooth muscle cells. Circ Res. 2009;105(3):249‐259. 19574552 10.1161/CIRCRESAHA.109.193722 PMC2744198

[39] Moreno MU , Gallego I , Lopez B , et al. Decreased Nox4 levels in the myocardium of patients with aortic valve stenosis. Clin Sci (Lond). 2013;125(6):291‐300. 23550626 10.1042/CS20120612

[40] Sutliff RL , Hilenski LL , Amanso AM , et al. Polymerase delta interacting protein 2 sustains vascular structure and function. Arterioscler Thromb Vasc Biol. 2013;33(9):2154‐2161. 23825363 10.1161/ATVBAHA.113.301913 PMC3837414

[41] Kim Y , Park H , Nah J , et al. Essential role of POLDIP2 in Tau aggregation and neurotoxicity via autophagy/proteasome inhibition. Biochem Biophys Res Commun. 2015;462(2):112‐118. 25930997 10.1016/j.bbrc.2015.04.084

[42] Papadopoulos MC , Manley GT , Krishna S , Verkman AS . Aquaporin‐4 facilitates reabsorption of excess fluid in vasogenic brain edema. FASEB J. 2004;18(11):1291‐1293. 15208268 10.1096/fj.04-1723fje

[43] Tait MJ , Saadoun S , Bell BA , Verkman AS , Papadopoulos MC . Increased brain edema in aqp4‐null mice in an experimental model of subarachnoid hemorrhage. Neuroscience. 2010;167(1):60‐67. 20132873 10.1016/j.neuroscience.2010.01.053 PMC2852644

[44] Cao C , Yu X , Liao Z , et al. Hypertonic saline reduces lipopolysaccharide‐induced mouse brain edema through inhibiting aquaporin 4 expression. Crit Care. 2012;16(5):R186. 23036239 10.1186/cc11670 PMC3682288

[45] Du KX , Dong Y , Zhang Y , et al. Effects of dexamethasone on aquaporin‐4 expression in brain tissue of rat with bacterial meningitis. Int J Clin Exp Pathol. 2015;8(3):3090‐3096. 26045822 PMC4440131

[46] Fortunato RS , Braga WM , Ortenzi VH , et al. Sexual dimorphism of thyroid reactive oxygen species production due to higher NADPH oxidase 4 expression in female thyroid glands. Thyroid. 2013;23(1):111‐119. 23033809 10.1089/thy.2012.0142

[47] Liu ZP , Wang Z , Yanagisawa H , Olson EN . Phenotypic modulation of smooth muscle cells through interaction of Foxo4 and myocardin. Dev Cell. 2005;9(2):261‐270. 16054032 10.1016/j.devcel.2005.05.017

[48] Essers MA , Weijzen S , de Vries‐Smits AM , et al. FOXO transcription factor activation by oxidative stress mediated by the small GTPase Ral and JNK. EMBO J. 2004;23(24):4802‐4812. 15538382 10.1038/sj.emboj.7600476 PMC535088

[49] Storz P , Doppler H , Copland JA , Simpson KJ , Toker A . FOXO3a promotes tumor cell invasion through the induction of matrix metalloproteinases. Mol Cell Biol. 2009;29(18):4906‐4917. 19564415 10.1128/MCB.00077-09 PMC2738298

[50] Diebold I , Petry A , Burger M , Hess J , Gorlach A . NOX4 mediates activation of FoxO3a and matrix metalloproteinase‐2 expression by urotensin‐II. Mol Biol Cell. 2011;22(22):4424‐4434. 21965295 10.1091/mbc.E10-12-0971 PMC3216667

[51] Li H , Liang J , Castrillon DH , DePinho RA , Olson EN , Liu ZP . FoxO4 regulates tumor necrosis factor alpha‐directed smooth muscle cell migration by activating matrix metalloproteinase 9 gene transcription. Mol Cell Biol. 2007;27(7):2676‐2686. 17242183 10.1128/MCB.01748-06 PMC1899894

[52] Kikuchi DS , Campos ACP , Qu H , et al. Poldip2 mediates blood‐brain barrier disruption in a model of sepsis‐associated encephalopathy. J Neuroinflammation. 2019;16(1):241. 31779628 10.1186/s12974-019-1575-4 PMC6883676

[53] Li Y , Wang X , Wang X , et al. PDCD4 suppresses proliferation, migration, and invasion of endometrial cells by inhibiting autophagy and NF‐kappaB/MMP2/MMP9 signal pathway. Biol Reprod. 2018;99(2):360‐372. 29912279 10.1093/biolre/ioy052

[54] Zhang JF , Wang P , Yan YJ , et al. IL33 enhances glioma cell migration and invasion by upregulation of MMP2 and MMP9 via the ST2‐NF‐kappaB pathway. Oncol Rep. 2017;38(4):2033‐2042. 28849217 10.3892/or.2017.5926 PMC5652951

[55] Sun L , Xu Q , Zhang W , Jiao C , Wu H , Chen X . The involvement of spinal annexin A10/NF‐kappaB/MMP‐9 pathway in the development of neuropathic pain in rats. BMC Neurosci. 2019;20(1):28. 31208343 10.1186/s12868-019-0513-9 PMC6580616

[56] Li X , Bao C , Ma Z , et al. Perfluorooctanoic acid stimulates ovarian cancer cell migration, invasion via ERK/NF‐kappaB/MMP‐2/‐9 pathway. Toxicol Lett. 2018;294:44‐50. 29753068 10.1016/j.toxlet.2018.05.009

[57] Chandrasekar B , Mummidi S , Mahimainathan L , et al. Interleukin‐18‐induced human coronary artery smooth muscle cell migration is dependent on NF‐kappaB‐ and AP‐1‐mediated matrix metalloproteinase‐9 expression and is inhibited by atorvastatin. J Biol Chem. 2006;281(22):15099‐15109. 16554298 10.1074/jbc.M600200200

[58] Frigeri A , Nicchia GP , Nico B , et al. Aquaporin‐4 deficiency in skeletal muscle and brain of dystrophic mdx mice. FASEB J. 2001;15(1):90‐98. 11149896 10.1096/fj.00-0260com

[59] Rurak J , Noel G , Lui L , Joshi B , Moukhles H . Distribution of potassium ion and water permeable channels at perivascular glia in brain and retina of the Large(myd) mouse. J Neurochem. 2007;103(5):1940‐1953. 17803675 10.1111/j.1471-4159.2007.04886.x

[60] Michele DE , Barresi R , Kanagawa M , et al. Post‐translational disruption of dystroglycan‐ligand interactions in congenital muscular dystrophies. Nature. 2002;418(6896):417‐422. 12140558 10.1038/nature00837

[61] Tham DK , Joshi B , Moukhles H . Aquaporin‐4 cell‐surface expression and turnover are regulated by dystroglycan, dynamin, and the extracellular matrix in astrocytes. PLoS One. 2016;11(10):e0165439. 27788222 10.1371/journal.pone.0165439 PMC5082936

[62] Noell S , Wolburg‐Buchholz K , Mack AF , et al. Evidence for a role of dystroglycan regulating the membrane architecture of astroglial endfeet. Eur J Neurosci. 2011;33(12):2179‐2186. 21501259 10.1111/j.1460-9568.2011.07688.x PMC3342013

[63] Warth A , Mittelbronn M , Wolburg H . Redistribution of the water channel protein aquaporin‐4 and the K+ channel protein Kir4.1 differs in low‐ and high‐grade human brain tumors. Acta Neuropathol. 2005;109(4):418‐426. 15723236 10.1007/s00401-005-0984-x

[64] Zhan Y , Tremblay MR , Melian N , Carbonetto S . Evidence that dystroglycan is associated with dynamin and regulates endocytosis. J Biol Chem. 2005;280(18):18015‐18024. 15728588 10.1074/jbc.M409682200

