# Supplementary material for: Poldip2 mediates blood‐brain barrier disruption and cerebral edema by inducing AQP4 polarity loss in mouse bacterial meningitis model
Source: CNS Neurosci Ther. 2020 Aug 12;26(12):1288–302. doi: 10.1111/cns.13446 (PMC7702237; doi:10.1111/cns.13446)

Full unedited blots for Figure 1

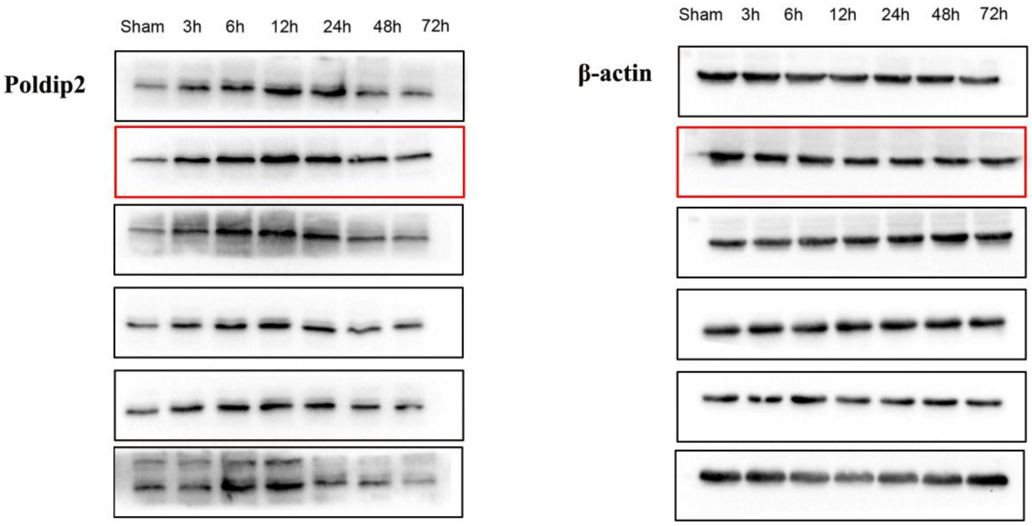

Full unedited blots for Figure 2

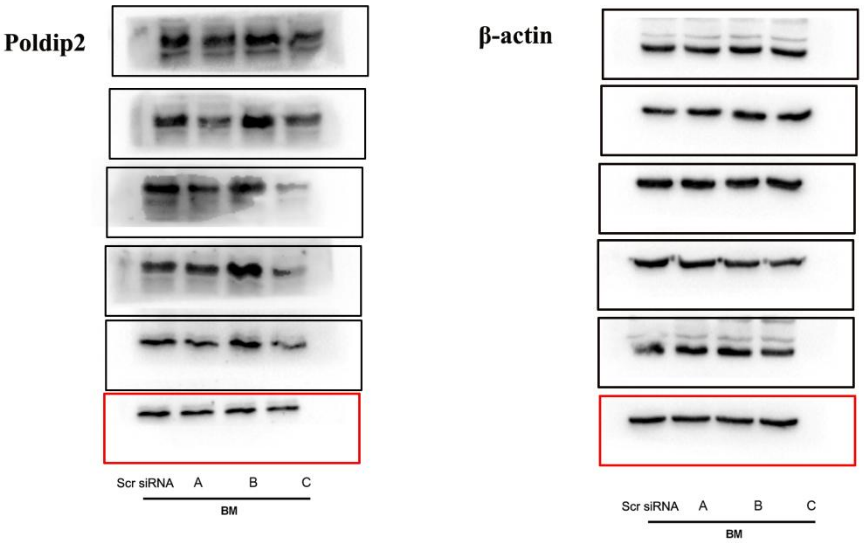

Full unedited blots for Figure 5

Poldip2

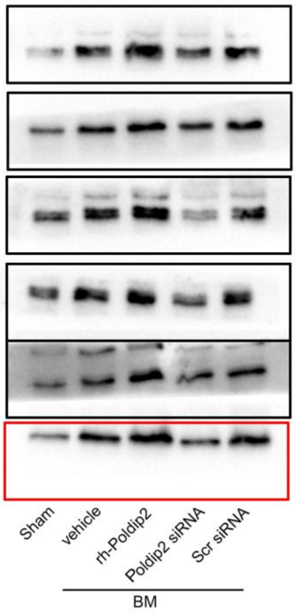

MMP9

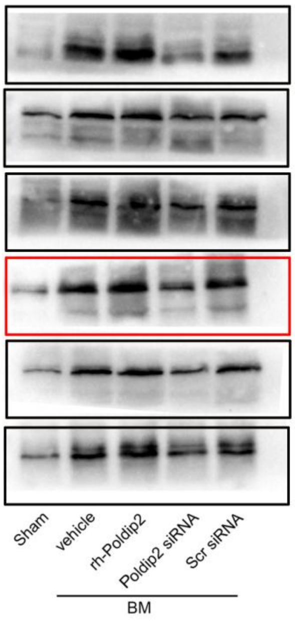

MMP2

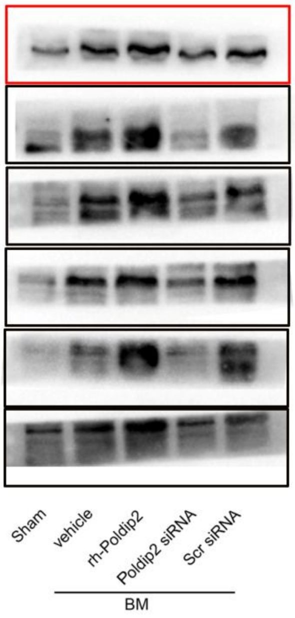

β-DG

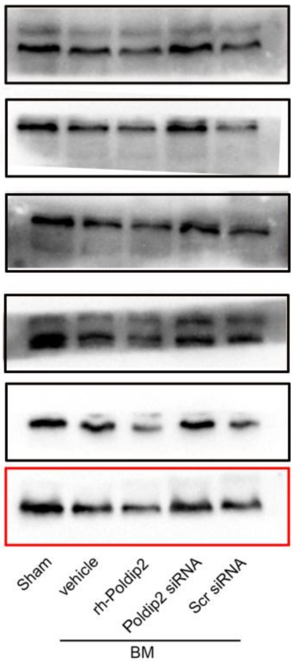

Full unedited blots for Figure 5

ZO-1

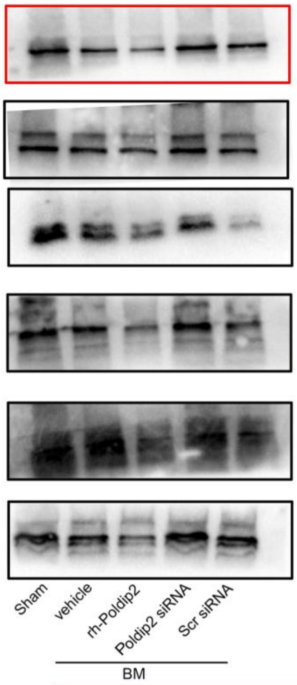

Occludin

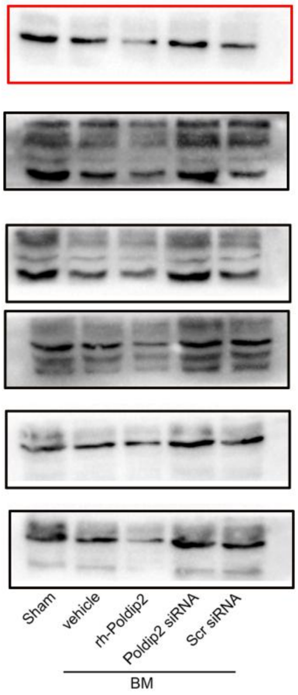

claudin-5

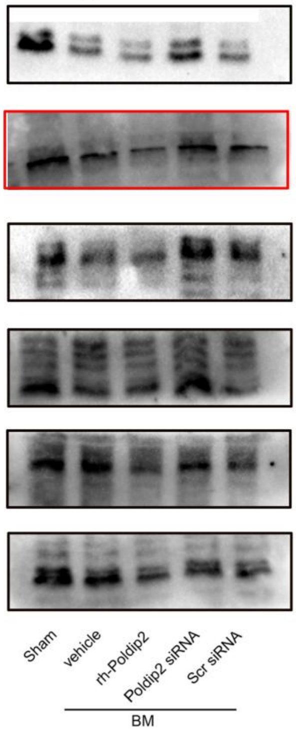

GFAP

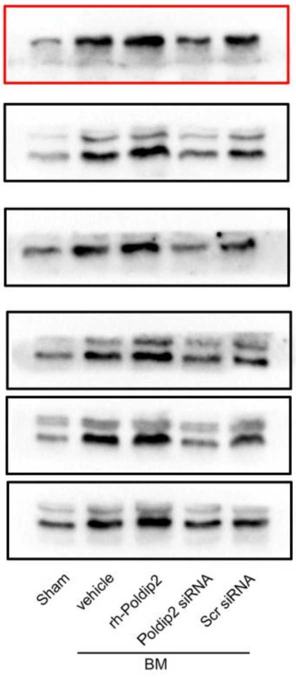

Full unedited blots for Figure 5

β-actin

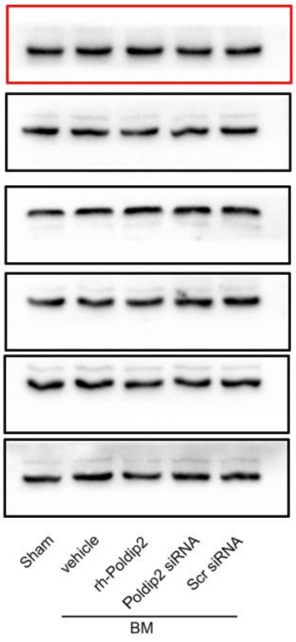

GAPDH

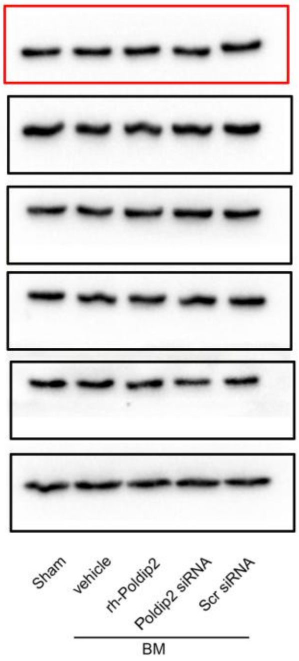

Full unedited blots for Figure 7

β-DG

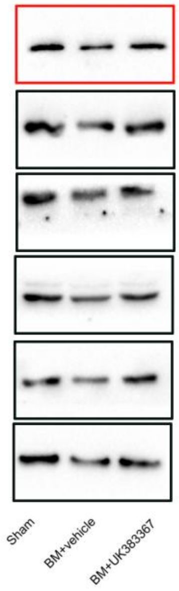

ZO-1

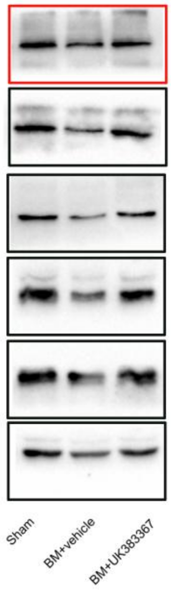

Full unedited blots for Figure 7

Occludin

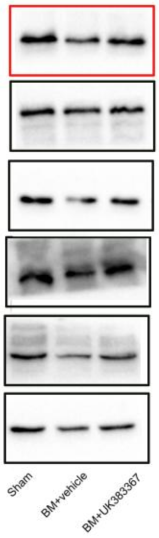

Claudin-5

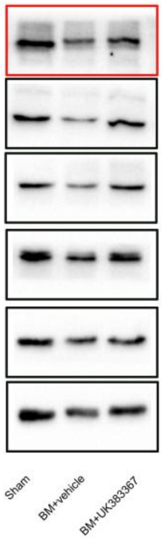

GFAP

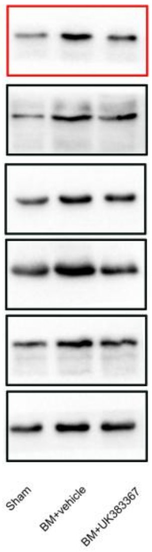

GAPDH

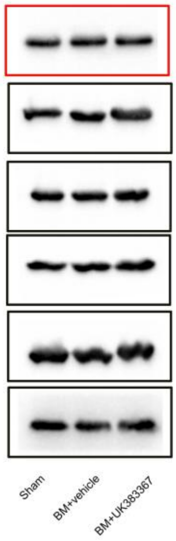

Full unedited blots for Figure 7

β-actin

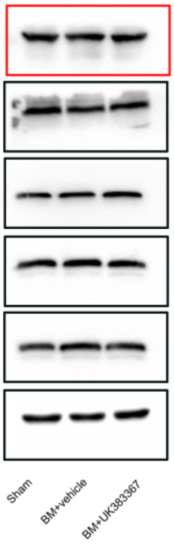

Supplement: Supplementary file 1 — Appendix S1 [file CNS-26-1288-s001.pdf]
